# Impact of polymorphisms of pharmacokinetics‐related genes and the inflammatory response on the metabolism of voriconazole

**DOI:** 10.1002/prp2.935

**Published:** 2022-02-23

**Authors:** Naoya Aiuchi, Junichi Nakagawa, Hirotake Sakuraba, Takenori Takahata, Kosuke Kamata, Norihiro Saito, Kayo Ueno, Masahiro Ishiyama, Kazufumi Yamagata, Hiroyuki Kayaba, Takenori Niioka

**Affiliations:** ^1^ Department of Pharmacy Hirosaki University Hospital Hirosaki Aomori Japan; ^2^ Department of Gastroenterology and Hematology Hirosaki University Graduate School of Medicine Hirosaki Aomori Japan; ^3^ Department of Clinical Laboratory Medicine Hirosaki University Graduate School of Medicine Hirosaki Aomori Japan; ^4^ Department of Clinical Laboratory Hirosaki University Hospital Hirosaki Aomori Japan; ^5^ Department of Bioscience and Laboratory Medicine Hirosaki University Graduate School of Health Sciences Hirosaki Japan; ^6^ Department of Pharmaceutical Science Hirosaki University Graduate School of Medicine Hirosaki Aomori Japan

**Keywords:** CRP, CYP2C19, *NR1I2* polymorphism, voriconazole, voriconazole *N*‐oxide

## Abstract

The effects of inflammatory responses and polymorphisms of the genes encoding cytochrome P450 (CYP) (*CYP2C19* and *CYP3A5*), flavin‐containing monooxygenase 3 (*FMO3*), pregnane X receptor (*NR1I2*), constitutive androstane receptor (*NR1I3*), and CYP oxidoreductase (*POR*) on the ratio of voriconazole (VRCZ) *N*‐oxide to VRCZ (VNO/VRCZ) and steady‐state trough concentrations (C_0h_) of VRCZ were investigated. A total of 56 blood samples were collected from 36 Japanese patients. Results of multiple linear regression analyses demonstrated that the presence of the extensive metabolizer *CYP2C19* genotype, the dose per administration, and the presence of the *NR1I2* rs3814057 C/C genotype were independent factors influencing the VNO/VRCZ ratio in patients with CRP levels of less than 40 mg/L (standardized regression coefficients (SRC) = 0.448, −0.301, and 0.390, respectively; all *p* < .05). With regard to the concentration of VRCZ itself, in addition to the above factors, the presence of the *NR1I2* rs7643645 G/G and rs3814055 T/T genotypes were found to be independent factors influencing the VRCZ C_0h_ in these patients (SRC = −0.430, 0.424, −0.326, 0.406 and −0.455, respectively; all *p* < .05). On the contrary, in patients with CRP levels of at least 40 mg/L, no independent factors were found to affect VNO/VRCZ and VRCZ C_0h_. Inflammatory responses, and *CYP2C19* and *NR1I2* polymorphisms may be useful information for the individualization of VRCZ dosages.

## Graphical abstract

Both the *CYP2C19* polymorphism and the status of the inflammatory response affect the *N*‐oxidation pathway, the major metabolic pathway of voriconazole. Therefore, the usefulness of *CYP2C19* genotype analysis in the individualization of voriconazole dosages depends on the state of the patient's inflammatory response. In addition, the *NR1I2* rs3814057 polymorphisms may also be useful in predicting voriconazole *N*‐oxide/voriconazole.

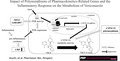

## 1 INTRODUCTION

Voriconazole (VRCZ) is widely used for the treatment and prophylaxis of a variety of invasive fungal diseases.^1^, ^2^ Complicating the use of this commonly used drug is the fact that it exhibits non‐linear pharmacokinetics with wide inter‐ and intra‐individual variability. The resulting low and high steady‐state trough concentrations (C_0h_) (< 1 µg/mL and > 4 to 6 µg/mL) are associated with treatment failure and serious adverse effects such as neurotoxicity and hepatotoxicity.^3^, ^4^, ^5^ Therefore, therapeutic drug monitoring (TDM) has been suggested as a strategy to optimize both the efficacy and safety of VRCZ.^6^, ^7^, ^8^

VRCZ is metabolized predominantly by cytochrome P450 (CYP) 2C19.^3^ CYP2C19 is primarily responsible for the conversion of VRCZ into its major inactive metabolite, VRCZ *N*‐oxide (VNO), which accounts for about 72% of plasma metabolites.^9^, ^10^ The genotype at the *CYP2C19* locus does contribute to the variation observed in VRCZ pharmacokinetics and potentially could be used to guide initial dose selections.^11^, ^12^ The variant alleles CYP2C19*2 and CYP2C19*3 are associated with decreased activity and thus higher C_0h_, while CYP2C19*17 is associated with increased activity.^13^ On the basis of the ability to metabolize CYP2C19 substrates, an individual can be classified as an ultra‐rapid metabolizer (UM; e.g., with genotype *1/*17 or *17/*17), extensive metabolizer (EM; *1/*1), intermediate metabolizer (IM; *1/*2 or *1/*3), or poor metabolizer (PM; *2/*2, *2/*3, or *3/*3). The distribution of *CYP2C19* polymorphisms varies among ethnic groups; approximately 20% of people in East Asian populations are classified as PM, which is higher than that in European Caucasian and African populations (less than 5%).^13^ Dosing recommendations for VRCZ based on CYP2C19 metabolizer type have been developed and are available from the Clinical Pharmacogenetics Implementation Consortium.^14^

It has been estimated that approximately 70 to 75% of total VRCZ metabolism is mediated through CYP enzymes and that 20 to 30% of this metabolism is through CYP3A4.^3^, ^10^, ^11^ However, proteins other than CYP must also be considered when investigating the pharmacokinetics of VRCZ. Most notably, it has been estimated that approximately 25 to 30% of total VRCZ metabolism is mediated through enzymes of the flavin‐containing monooxygenase (FMO) family.^15^ Therefore, genetic heterogeneity in these genes may influence interindividual variability of VRCZ C_0h_.^16^, ^17^

Three other genes that potentially impact VRCZ metabolism are *POR*, *NR1I2*, and *NR1I3*. *POR* encodes CYP oxidoreductase, which increases the turnover of CYP substrates by providing electrons to the CYP enzyme to activate its reductive activity.^18^ *NR1I2* and *NR1I3* both encode nuclear receptors that lead to the induction of expression of multiple genes that encode metabolic enzymes, including CYP.^19^ Specifically, *NR1I2* encodes the pregnane X receptor (PXR), and *NR1I3* encodes the constitutive androstane receptor (CAR).

A possible insight into the prediction of variations of VRCZ pharmacokinetics has come with recent studies that have reported positive correlations between C‐reactive protein (CRP) levels and VRCZ C_0h_,^20^, ^21^, ^22^, ^23^, ^24^, ^25^, ^26^, ^27^, ^28^ which suggests a connection between VRCZ metabolism and inflammatory responses. However, how inflammatory status modulates the impact of polymorphisms of other pharmacokinetics‐related genes, such as *NR1I2*, *NR1I3*, *POR*, and *FMO3*, which may affect a metabolic pathway from VRCZ to VNO, remains unknown.

The purpose of this study, therefore, was to analyze the effect of *CYP2C19* polymorphisms combined with the inflammatory status on the VRCZ metabolic ratio (VNO/VRCZ). In addition, as several single nucleotide polymorphisms (SNPs) of *CYP3A5*, *FMO3*, *NR1I2*, *NR1I3*, and *POR* have been associated with high expression of these genes in the Japanese population, contributions of these sites to VNO/VRCZ and VRCZ C_0h_ were also investigated.

## 2 MATERIALS AND METHODS

### 2.1 Ethics

This study was carried out at a single institution. The study was conducted in accordance with the Declaration of Helsinki, and the protocol was approved by the Ethics Committee of Hirosaki University Graduate School of Medicine (project identification code: 2019–1021, 2019–1140). Informed consent was obtained from all patients enrolled in this study.

### 2.2 Patients and sample collection

Japanese patients receiving VRCZ (Vfend^®^, Pfizer Japan Inc.) therapy for prophylaxis or treatment of fungal infection and who were subjected to TDM testing at Hirosaki University Hospital from January 2018 to July 2021 were enrolled in this study. TDM data of patients taking VRCZ were collected retrospectively. Patients were excluded if they (a) were aged less than 18 years; (b) were receiving dialysis; (c) did not undergo genotyping; (d) were taking drugs known to affect VRCZ pharmacokinetics.^29^ Although some patients were taking proton pump inhibitors (PPIs), they were included in this analysis because of the weak effect of PPIs on VRCZ pharmacokinetics.^30^

Multiple blood levels of VRCZ from the same patient were utilized in the analyses when the following patients’ status changed during treatment: (a) inflammatory status (CRP ≥ 40 mg/L or < 40 mg/L); (b) route of administration of VRCZ (intravenous or oral); (c) dose of VRCZ per time. Each patient received 100 to 300 mg of VRCZ twice daily at 10:00 and 22:00 for at least 3 days. VRCZ was administered between meals to patients who were eligible for oral administration. Initial oral or intravenous doses were determined according to the physician’s clinical judgment. The target C_0h_ of VRCZ was 1 to 5 µg/mL.^5^ Demographic data, including age, sex, body weight, and liver and renal function tests, were collected. For age and body weight, the values at the initiation of VRCZ therapy were considered. For laboratory values, such as CRP, the values on the day TDM of VRCZ was carried out were considered.

Blood samples within 50 days after initiation of VRCZ therapy were collected in disodium EDTA tubes. These samples were collected just prior to the subsequent dosage when the plasma concentration would be at a steady state. After dose adjustment, a 3‐day period was considered necessary to obtain a VRCZ C_0h_ steady state.^31^ The VNO/VRCZ was calculated using C_0h_ values of the two compounds.

### 2.3 Methods of analysis of plasma concentrations

Blood samples were centrifuged at 3500 rpm for 10 min at 4°C, and separated plasma was stored at −30°C until analysis. Plasma concentrations of VRCZ and VNO were measured by ultra‐performance liquid chromatography (UPLC) tandem mass spectrometry using an ACQUITY UPLC System (Waters, MA, USA). Plasma (100 μL) was mixed with 150 μL of acetonitrile and 10 μL of 10 μg/mL VRCZ‐d3 as an internal standard. The mixture was vortexed for 30 s and centrifuged at 13500 rpm for 5 min at room temperature. A fraction of the supernatant (100 μL) was diluted with 200 μL MilliQ water (total volume: 300 μL). The sample was transferred to an autosampler vial, and 2 μL was injected into an ACQUITY UPLC HSS C18 column (1.8 µm, 2.1 mm × 100 mm) at 40°C. The mobile phase consisted of MilliQ water with 0.1% formic acid (A) and acetonitrile with 0.1% formic acid (B) at a flow rate of 0.4 mL/min. Gradient conditions were as follows: constant 5% B from 0 to 1.0 min; linear increase from 5% to 95% from 1.0–6.0 min; constant 95% B from 6.0 to 7.0 min; linear decrease from 95% to 5% B from 7.0 to 7.1 min; and constant 5% B from 7.1 to 10.0 min.

The analyte and internal standard were ionized and detected using a Xevo TQD (Waters). Positive electrospray ionization was performed in the multiple reaction monitoring mode. Ion transitions (in m/z) of VRCZ, VNO, and internal standard were 350.2 to 281.1, 366.0 to 224.0, and 352.9 to 283.8, respectively. The cone voltage and collision energies were 30 V and 30 eV for VRCZ, 30V and 15 eV for VNO, and 30V and 20 eV for the internal standard. The calibration curve was linear in the range of 0.25 to 10 μg/mL. The calibration curve showed good linearity, with *R* ^2^ > 0.99. The intra‐ and inter‐day accuracy values, expressed as percent CV, were all within ± 15%, and precision values (as percent CV) were all less than 15% in each calibration curve.

### 2.4 Genotyping

DNA was extracted from peripheral blood samples with a QIAamp Blood Kit (Qiagen, Hilden, Germany) and was stored at −30°C until analysis. The following genotypes were determined by real‐time PCR using TaqMan SNP Genotyping Assays from Thermo Fisher Scientific (Waltham, MA, USA): CYP3A5*3 (rs776746, c.6986A>G), CYP2C19*2 (rs4244285, c.681G>A), CYP2C19*3 (rs4986893, c.636G>A), CYP2C19*17 (rs12248560, c.‐806C>T), *NR1I3* (rs2307424, c.540C>T), *NR1I2* (rs3814057, A>C), *NR1I2* (rs7643645, A>G), *NR1I2* (rs2472677, C>T), *NR1I2* (rs3814055, C>T), POR*28 (rs1057868, G>A), *FMO3* (rs2266780, A>G), and *FMO3* (rs2266782, G>A). Cycle conditions were as follows: initial hold at 50°C for 2 min and hold at 95°C for 10 min, followed by 40 cycles of 95°C for 15 s and 60°C for 30 s. Genotypes were detected using a CFX‐Connect Real‐Time PCR system (Bio‐Rad Laboratories Inc., Hercules, CA, USA).

### 2.5 Statistical analysis

The Shapiro‐Wilk test was used to assess distribution. Descriptive statistics of continuous variables are presented as mean ± standard deviation (SD), minimum and maximum, or median (interquartile range). Allele frequencies of polymorphisms were evaluated according to the Hardy‐Weinberg equilibrium using χ^2^ tests. The Kruskal–Wallis test or Mann–Whitney U test was used to determine differences in continuous values between groups. The Spearman’s rank correlation coefficient test was used to assess correlations in continuous values between groups, and these results were expressed as Spearman’s *ρ* values. Analysis of variance was performed to evaluate the relationships between *CYP2C19* enotypes and CRP in their influence on VNO/VRCZ. Multiple linear regression analyses in which *CYP2C19* genotypes and CRP were forced entries were performed to identify the factors that influence the VNO/VRCZ ratio and VRCZ C_0h_ from all factors in a univariate analysis. Among all possible regression models, a multiple regression equation was adopted in which significant differences were found in all of the explanatory variables and a coefficient of determination (*R^2^*) was the highest. Each genotype was replaced with dummy variables (1 and 0, 0 and 1, and 0 and 0, respectively). The results with *p*‐values of less than .05 were considered statistically significant. Statistical analyses were performed with SPSS 27.0 for Windows (SPSS IBM Japan Inc., Tokyo, Japan).

## 3 RESULTS

The demographic and clinical information of the patients at the time of the TDM are listed in Table 1. A total of 56 blood samples from 36 patients, ranging from 1 to 3 samples per patient, were obtained. Blood samples for TDM of VRCZ were collected between 4 and 50 days after the start of administration. According to the exclusion criteria, patients taking erythromycin (n = 2) or clarithromycin (n = 2) were excluded from the study because these drugs have been demonstrated to affect VRCZ pharmacokinetics.^29^ None of the patients in this study had severe liver dysfunction, as defined by a Child‐Pugh score of B or C. Thirty patients were taking PPIs; 24 of these patients were taking lansoprazole.

**TABLE 1:** Patient demographic and clinical characteristics

Parameter	Mean ± SD	Range
Age (yr)	67.2 ± 14.1	19–96
Body weight (kg)	55.9 ± 14.3	37.5–103.8
Laboratory values		
AST (U/L)	39.2 ± 38.3	9–204
ALT (U/L)	31.4 ± 39.1	6–240
T‐Bil (mg/L)	6.2 ± 7.1	2–40
eCCr (mL/min)	76.9 ± 39.2	14.1–157.0

	Median	Interquartile range
CRP (mg/L)	11.0	1.1–76.6
		**n**
Route of administration		
Intravenous:Oral		24:32
Sex		
Male:Female		29:27
PPI coadministration		
Yes:No		30:26
OPZ:EPZ:LPZ:RPZ		1:2:24:3
Genotypes		
*CYP2C19*		
*1/*1:*1/*2:*1/*3:*2/*2:*2/*3:*3/*3		15:17:7:7:8:2
*CYP3A5*		
*1/*1:*1/*3:*3/*3		4:20:32
*NR1I3* rs2307424 C>T		
C/C:C/T:T/T		8:27:21
*NR1I2* rs3814057 A>C		
A/A:A/C:C/C		18:23:15
*NR1I2* rs7643645 A>G		
A/A:A/G:G/G		22:25:9
*NR1I2* rs2472677 C>T		
C/C:C/T:T/T		4:32:20
*NR1I2* rs3814055 C>T		
C/C:C/T:T/T		23:30:3
*POR* rs1057868 C>T		
C/C:C/T:T/T		20:25:11
*FMO3* rs2266780 A>G (rs2266782 G>A)		
A/A:A/G (G/G:G/A)		35:21

Abbreviations: ALT, alanine aminotransferase; AST, aspartate aminotransferase; CRP, C‐reactive protein; eCCr, estimated creatinine clearance; EPZ, esomeprazole; LPZ, lansoprazole; OPZ, omeprazole; PPI, proton pump inhibitor; RPZ, rabeprazole; T‐Bil, total bilirubin.

eCCr = [(140 ‐ age) × body weight (kg)] / [72 × serum creatinine (mg/dL)] (× 1.0 for male or × 0.85 for female)

All of the SNPs (*CYP2C19*; *CYP3A5*; *NR1I3* rs2307424; *NR1I2* rs3814057, rs7643645, rs2472677, and rs3814055; *POR* rs1057868; and *FMO3* rs2266780, rs2266782) were in Hardy‐Weinberg equilibrium (*p* = .994, .801, .876, .628, .969,.341,.251, .994, .417, and .417, respectively). None of the patients had the CYP2C9*17 allele. There was complete linkage between the polymorphisms leading to the E158K and E308G mutations in FMO3.

**FIGURE 1:**
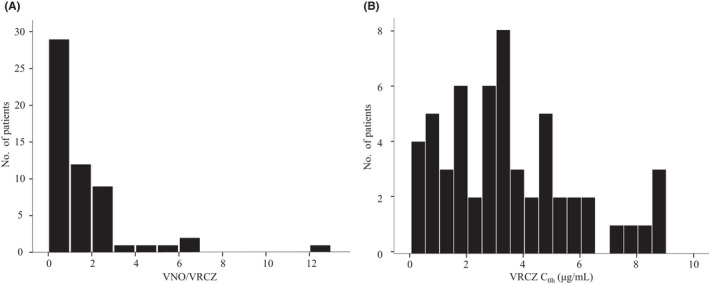
Histograms of (A) VNO/VRCZ and (B) VRCZ C_0h_. VRCZ, voriconazole; VNO/VRCZ, ratio of VRCZ *N*‐oxide of VRCZ; C_0h_, steady‐state trough concentrations of VRCZ

**FIGURE 2:**
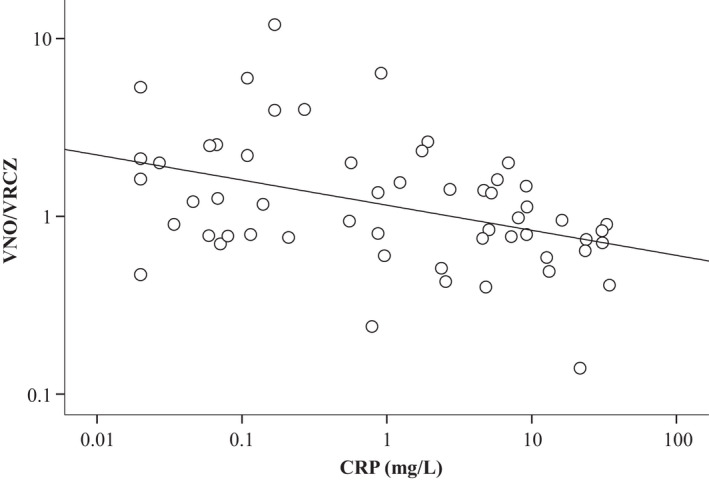
Relationships of VNO/VRCZ to inflammation status. The concentrations of voriconazole (VRCZ), VRCZ *N*‐oxide (VNO), and C‐reactive protein (CRP) in patient blood plasma were determined as described in Materials and Methods

No normality was found in the distributions of the VNO/VRCZ ratio and VRCZ C_0h_ (Figure 1). VRCZ C_0h_ values outside the therapeutic range (C_0h_ < 1.0 µg/mL, n = 9, and C_0h_ > 5.0 µg/mL, n = 12) accounted for 37.5% of the total. As shown in Figure 2, there was a significant correlation between VNO/VRCZ ratios and levels of CRP (*ρ* = −0.404, *p* = .002). Accordingly, there were significant differences in median VNO/VRCZ ratios between patients with CRP levels of less than 40 mg/L and patients with CRP levels of at least 40 mg/L (1.39 vs. 0.81, *p *= .007) (Table 2). Differences in median VNO/VRCZ ratios were not significant among *CYP2C19* genotype groups (CYP2C19 EM:IM:PM ratios = 1.55:0.95:0.80, *p* = .191) (Table 2).

**TABLE 2:** Comparisons of VNO/VRCZ and VRCZ C_0h_ upon plasma concentration monitoring after VRCZ administration relative to patient demographic and clinical characteristics

	VNO/VRCZ			VRCZ C_0h_ (µg/mL)		
	Median	(Interquartile range)	*p*‐value	Median	(Interquartile range)	*p*‐value
Sex			.020			.005
Female	1.35	(0.80–2.57)		2.9	(1.2–3.6)	
Male	0.80	(0.60–1.42)		4.1	(2.8–3.6)	
Route of administration			.043			.403
Intravenous	0.80	(0.72–1.28)		3.3	(2.5–5.2)	
Oral	1.39	(0.79–2.15)		3.2	(1.5–4.4)	
PPI coadministration			.761			.297
Yes	1.06	(0.74–2.00)		3.1	(1.7–4.1)	
No	0.92	(0.75–2.00)		3.4	(1.9–5.8)	
CRP			.007			.104
<40 mg/L	1.39	(0.78–2.50)		2.9	(0.9–4.6)	
≥40 mg/L	0.81	(0.64–1.13)		3.4	(2.9–4.9)	
*CYP2C19* genotypes			.191			.434
*1/*1	1.55	(0.95–2.98)		2.8	(1.7–3.6)	
*1/*2 + *1/*3	0.95	(0.77–1.81)		3.4	(1.8–5.6)	
*2/*2 + *2/*3 + *3/*3	0.80	(0.60–1.26)		3.2	(2.5–5.0)	
*CYP3A5* genotypes			.576			.421
*1/*1	1.54	(0.99–1.80)		4.0	(3.1–6.7)	
*1/*3	1.04	(0.76–2.35)		3.0	(1.1–5.2)	
*3/*3	0.92	(0.70–1.59)		3.3	(1.9–4.6)	
*NR1I3* rs2307424 C>T			.617			.644
C/C	0.78	(0.60–3.27)		4.7	(1.7–6.8)	
C/T	0.90	(0.75–1.70)		3.2	(1.6–4.4)	
T/T	1.36	(0.84–1.62)		3.1	(2.5–4.6)	
*NR1I2* rs3814057 A>C			.057			.225
A/A	0.80	(0.59–1.62)		3.2	(2.8–5.1)	
A/C	0.95	(0.74–1.52)		3.5	(2.0–4.8)	
C/C	1.40	(1.00–3.24)		2.0	(1.2–3.5)	
*NR1I2* rs7643645 A>G			.200			.994
A/A	0.89	(0.64–1.55)		3.2	(2.0–4.6)	
A/G	1.26	(0.84–2.00)		3.3	(1.8–4.6)	
G/G	0.75	(0.49–2.33)		2.9	(2.0–5.1)	
*NR1I2* rs2472677 C>T			.594			.839
C/C	2.45	(0.72–7.98)		5.4	(1.2–8.8)	
C/T	1.15	(0.77–1.81)		3.4	(1.6–4.8)	
T/T	0.89	(0.62–1.78)		2.9	(2.0–4.4)	
*NR1I2* rs3814055 C>T			.664			.345
C/C	1.26	(0.75–2.35)		3.2	(1.4–5.0)	
C/T	0.97	(0.76–1.42)		3.4	(2.0–4.6)	
T/T	0.80	(0.69–1.57)		2.5	(1.6–2.7)	
*POR* rs1057868 C>T			.800			.802
C/C	0.95	(0.77–2.35)		3.2	(1.3–5.4)	
C/T	1.35	(0.60–1.62)		2.9	(1.8–4.1)	
T/T	0.90	(0.78–1.19)		3.6	(2.7–4.6)	
*FMO3* rs2266780 A>G (rs2266782 G>A)			.326			.685
A/A (G/G)	1.17	(0.77–1.61)		3.3	(2.0–4.6)	
A/G (G/A)	0.80	(0.49–2.11)		3.0	(1.5–4.9)	

	Correlation coefficient (*ρ*)	*p*‐value	Correlation coefficient (*ρ*)	*p*‐value
Dose per administration (mg)	−0.13	.334	0.33	.014
Age (yr)	0.28	.039	−0.30	.023
Body weight (kg)	−0.38	.004	0.31	.017
Laboratory values				
AST (U/L)	−0.23	.085	0.19	.170
ALT (U/L)	−0.22	.098	0.27	.043
T‐Bil (mg/L)	−0.20	.183	0.06	.692
eCCr (mL/min)	−0.33	.014	0.35	.008

Abbreviations: ALT, alanine aminotransferase; AST, aspartate aminotransferase; CRP, C‐reactive protein; eCCr, estimated creatinine clearance; PPI, proton pump inhibitor; T‐Bil, total bilirubin.

eCCr = [(140 ‐ age) × body weight (kg)] / [72 × serum creatinine (mg/dL)] (× 1.0 for male or × 0.85 for female).

**FIGURE 3:**
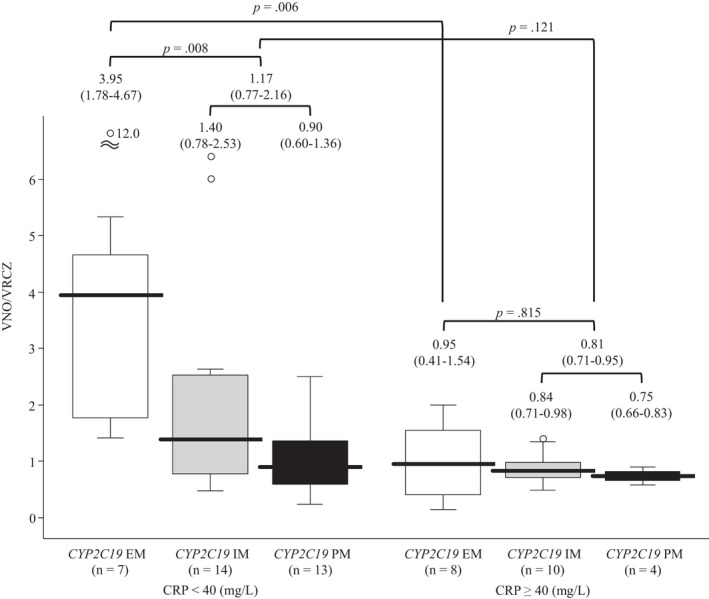
Comparison of VNO/VRCZ with respect to differences in inflammatory status and *CYP2C19* genotype. The concentrations of voriconazole (VRCZ), VRCZ *N*‐oxide (VNO), and C‐reactive protein (CRP) in patient blood plasma were determined as described in Materials and Methods. Patient genotypes are as follows: EM (extensive metabolizer), CYP2C19 *1/*1; IM (intermediate metabolizer), CYP2C19 *1/*2 or *1/*3; PM (poor metabolizer), CYP2C19 *2/*2 or *2/*3 or *3/*3. Box and whisker plots were prepared with SPSS. The box spans data between two quartiles (IQR, interquartile range), with the median represented as a bold horizontal line. The ends of the whiskers (vertical lines) represent the smallest and largest values that were not outliers. Outliers (Ο) are values between 1.5 and 3 IQRs from the end of the box. The values above the upper limits of the *y* axis are shown with a scale break line. The values are expressed as median (IQR)

Analysis of variance showed that *CYP2C19* EM and CRP levels less than 40 mg/L were independent factors influencing the VNO/VRCZ ratio (both *p* < .01) and that there was an interaction between them (*p* < .05). The median ratios of the VNO/VRCZ in patients with CRP levels less than 40 mg/L were different between *CYP2C19* EM patients and other *CYP2C19* genotype groups (IM and PM) (3.95 vs. 1.17, *p* = .008), but the median ratios in patients with CRP levels of at least 40 mg/L were not significantly different between those genotypes (0.95 vs. 0.81, *p* = .815) (Figure 3). On the other hand, the median ratios of the VNO/VRCZ in patients with the *CYP2C19* EM genotype were different between patients with CRP levels of less than 40 mg/L and those with CRP levels of at least 40 mg/L (*p* = .006), but the same comparison in patients with *CYP2C19* IM and PM genotypes did not indicate a significant difference (*p* = .121) (Figure 3).

**FIGURE 4:**
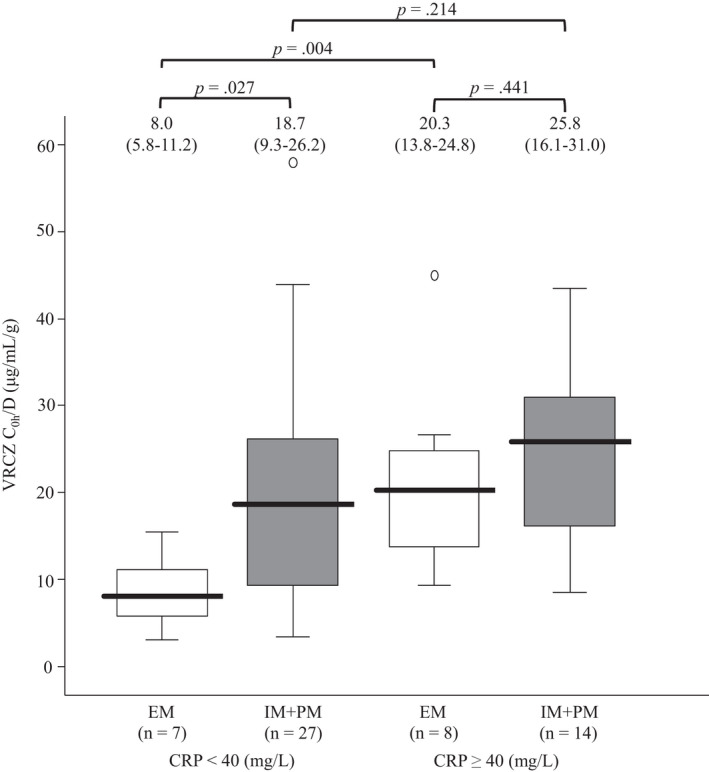
Comparison of C_0h_/D with respect to the differences in inflammatory status and *CYP2C19* genotype. VRCZ, voriconazole; VNO/VRCZ, ratio of VRCZ *N*‐oxide to VRCZ; C_0h_, steady‐state trough concentrations of VRCZ; C_0h_/D, VRCZ dose‐adjusted C_0h_; CRP, C‐reactive protein. EM (extensive metabolizer), CYP2C19 *1/*1; IM (intermediate metabolizer), CYP2C19 *1/*2 or *1/*3; PM (poor metabolizer), CYP2C19 *2/*2 or *2/*3 or *3/*3. Graphical analysis was performed using an SPSS box and whiskers plot. The box spans data between two quartiles (IQR, interquartile range ), with the median represented as a bold horizontal line. The ends of the whiskers (vertical lines) represent the smallest and largest values that were not outliers. Outliers (Ο) are values between 1.5 and 3 IQRs from the end of the box. The values above the upper limits of the *y* axis are shown with a scale break line. The values are expressed as median (IQR)

The median of the VRCZ dose‐adjusted C_0h_ (C_0h_/D) in patients with CRP levels less than 40 mg/L was different between patients with *CYP2C19* EM genotypes and those with *CYP2C19* IM and PM genotypes (8.0 vs. 18.7, *p* = .027), but this median in patients with CRP levels of at least 40 mg/L was not different between those genotypes (20.3 vs. 25.8, *p* = .441) (Figure 4). On the other hand, the median of the VRCZ C_0h_/D in patients with the *CYP2C19* EM genotype was different between patients with CRP levels of less than 40 mg/L and those with CRP levels of at least 40 mg/L (*p* = .004), but this median in patients with *CYP2C19* IM and PM genotypes was not significantly different (*p* = .214) (Figure 4).

Tables 2 and 3 show the influence of biological and clinical data on VNO/VRCZ and VRCZ C_0h_. Results of multiple linear regression analyses demonstrated that the presence of the *CYP2C19* EM genotype, the dose per administration, and the presence of the *NR1I2* rs3814057 C/C genotype were independent factors influencing the VNO/VRCZ ratio in patients with CRP levels of less than 40 mg/L (SRC = 0.448, −0.301, and 0.390, respectively; all *p* < .05). The final model explained 45.5% of the variability in VNO/VRCZ. With regard to the concentration of VRCZ itself, in addition to the above factors, the presence of the *NR1I2* rs7643645 G/G and rs3814055 T/T genotypes were found to be independent factors influencing the VRCZ C_0h_ in these patients (SRC = −0.430, 0.424, −0.326, 0.406, and −0.455, respectively; all *p* < .05). The final model explained 54.5% of the variability in VRCZ C_0h_. On the contrary, in patients with CRP levels of at least 40 mg/L, no independent factors were found to affect VNO/VRCZ and VRCZ C_0h_.

**TABLE 3:** Multiple regression analysis for VNO/VRCZ and VRCZ C_0h_

Dependent variable	Explanatory variable	Slope	SE	SRC	*p*‐value	*R* ^2^
VNO/VRCZ (patients with CRP levels of less than 40 mg/L, n = 34)						0.455
	*CYP2C19* genotype (*1/*1 = 1)	2.554	0.782	0.448	.003	
	Dose per administration (mg)	−0.006	0.003	−0.301	.034	
	*NR1I2* rs3814057 (C/C = 1)	1.975	0.690	0.390	.008	
		Intercept = 3.216	1.020			
VNO/VRCZ (patients with CRP levels of at least 40 mg/L, n = 22)						
		No independent factors				
VRCZ C_0h_ (patients with CRP levels of less than 40 mg/L, n = 34)						0.545
	*CYP2C19* genotype (*1/*1 = 1)	−2.471	0.762	−0.430	.003	
	Dose per administration (mg)	0.018	0.005	0.424	.003	
	*NR1I2* rs3814057 (C/C = 1)	−1.661	0.667	−0.326	.019	
	*NR1I2* rs7643645 (G/G = 1)	2.335	0.859	0.406	.011	
	*NR1I2* rs3814055 (T/T = 1)	−4.492	1.497	−0.455	.006	
		Intercept = 1.569	0.699			
VRCZ C_0h_ (patients with CRP levels of at least 40 mg/L, n = 22)						
		No independent factors				

Abbreviations

SE, standard error; SRC, standardized regression coefficient.

## 4 DISCUSSION

The results presented above suggest that the effects of *CYP2C19* polymorphisms on the VNO/VRCZ ratio may be modulated by the severity of inflammation reactions. Previous *in vitro* studies have demonstrated that proinflammatory cytokines, such as IL‐6, downregulate the expression of *CYP2C19* and *CYP3A4*.^32^, ^33^ Therefore, in patients experiencing inflammation, decreased levels of these CYP enzymes might cause an increase in VRCZ C_0h_. Accordingly, Vreugdenhil et al. reported that VRCZ C_0h_ correlates with IL‐6, IL‐8, and CRP levels.^22^ In addition, in a report by Gautier et al., both *CYP2C19* and *CYP3A4* genotypes and inflammation were shown to significantly influence VRCZ C_0h_.^23^

However, while these studies suggested quantitative connections among inflammation, the CYP enzymes, and VRCZ pharmacokinetics, VNO concentrations were not measured, so the impact of inflammation on the metabolic pathway was not clarified. In order to further investigate this connection, we investigated the effect of the inflammatory response on VNO/VRCZ and C_0h_/D. To this end, we used a CRP cutoff value (40 mg/L) that has been established in a previous report^28^ to differentiate the severity of inflammation. There were significant differences in VNO/VRCZ and C_0h_/D between patients with different *CYP2C19* genotypes only when the patients had CRP levels less than 40 mg/L, but not when the patients had CRP levels of at least 40 mg/L. In addition, inflammatory responses were found to decrease VNO/VRCZ and increase VRCZ C_0h_/D in patients with the *CYP2C19* EM genotype, but not in groups with the CYP2C19*2 or *3 alleles (i.e., the IM or PM genotypes). These results are consistent with the results of Gautier et al, who concluded that strategies for the individualization of VRCZ dose should integrate the inflammatory status of patients in addition to pharmacogenetic variants.^20^ In addition, these results are in accord with the results of Simon et al, who used simulations with physiologically based pharmacokinetic models of VRCZ and reported that inflammatory responses affected CYP2C19 activity strongly at intermediate CRP levels (20 to 40 mg/L).^34^ Thus, our results confirmed that in an actual TDM in a clinical setting, the effect of *CYP2C19* polymorphisms on CYP2C19 activity changed across this CRP range.

Here, in addition to *CYP2C19* polymorphisms, the dose per administration of VRCZ and the presence of the *NR1I2* rs3814057 polymorphism were also independent factors influencing the VNO/VRCZ ratio in patients with CRP levels of less than 40 mg/L, according to multivariate linear regression analyses stratified by CRP. Furthermore, in addition to these factors, the *NR1I2* rs7643645 and rs3814055 polymorphisms were independent factors influencing the VRCZ C_0h_ in these patients. PXR influences the activities of a variety of metabolic enzymes and transporters,^35^, ^36^ and it is thus not surprising that SNPs in the *NR1I2* gene would affect the activity of CYP2C19 and/or CYP3A4. Notably, whereas CYP2C19 is responsible for the main pathway leading to *N*‐oxidation, CYP3A4 appears to play a more important role in the hydroxylation pathway.^10^, ^37^ Therefore, it is possible that the effect of *NR1I2* polymorphisms on the VNO/VRCZ ratio and VRCZ C_0h_ also reflects an effect of CYP3A4 on VRCZ metabolism.

Our results were in contrast to the results of Zeng et al., who reported that the *NR1I2* rs3814057 C/C genotype increased VRCZ C_0h_ and the rs7643645 G/G genotype decreased VRCZ C_0h_ in an Asian population.^17^ This discrepancy may be explained by these authors’ lack of consideration of the effects of *CYP2C19* polymorphisms on VRCZ C_0h_.^17^ In other words, it is possible that these were confounding factors. On the other hand, the work of Dapía et al., who reported that the *NR1I2* rs3814055 T/T genotype decreased VRCZ AUC_0‐∞_ in a Spanish population,^16^ was consistent with our results. However, our work extends these results by also demonstrating the effect of the inflammatory response on VRCZ C_0h_.^16^ While other previous studies have shown definitively that both *CYP2C19* polymorphisms^38^, ^39^ and inflammation status^20^, ^21^, ^22^, ^23^, ^24^, ^25^, ^26^, ^27^, ^28^ affect blood levels of VRCZ, ours is the first study to show the complex impacts of *CYP2C19* and *NR1I2* polymorphisms and CRP levels on the pharmacokinetics of VRCZ.

In our study, the CYP3A5*3, *NR1I3*, *FMO3*, and POR*28 polymorphisms did not affect the plasma concentration of VRCZ. To our knowledge, there are no reports that CYP3A5*3 or *NR1I3* polymorphisms affected VRCZ pharmacokinetics. In fact, one previous report did not support the consideration of dose adjustments of VRCZ based on *CYP3A5* polymorphisms.^40^ In contrast, other results have been obtained for the effects of *FMO3* polymorphisms on the plasma concentration levels of VRCZ. In a univariate analysis of VRCZ pharmacokinetics‐pharmacogenetics in a Japanese population, the *FMO3* E158K/E308G (rs2266782/rs2266780) genotype was shown to affect the plasma concentration of VRCZ.^41^ In a similar univariate analysis in a Chinese population, the *FMO3* rs2266780 genotype was shown to affect the plasma concentration of VRCZ, but a relationship was not demonstrated upon a multivariate analysis.^17^ Conversely, in a multivariate analysis investigating VRCZ plasma concentrations in a Spanish population, the *FMO3* rs1800822 genotype was found to be an independent factor affecting the plasma concentration of VRCZ, but the *FMO3* rs2266782 genotype was not.^16^ Regarding the effect of the *POR* polymorphism on the plasma concentration of VRCZ, no consistent results have been obtained so far. Although A503V is a common amino acid sequence variant encoded by *POR* rs1057868,^42^ this genotype did not affect the VRCZ C_0h_ or the VNO/VRCZ in our study. In previous studies, although *POR* rs1057868 affected the plasma concentration of VRCZ in a Spanish population,^16^ no relationship was observed in a Chinese population.^17^ Importantly, other *POR* variants may also affect CYP‐mediated drug metabolism activities.^43^ Therefore, the relationships between the pharmacokinetics of VRCZ and the *FMO3* and *POR* polymorphisms need to be examined further.

This study had a few limitations. First, the number of patients included in our study population was small, so we may not have been able to definitively prove the effect of the *NR1I2* polymorphism on VRCZ pharmacokinetic characteristics in multivariate linear regression analyses stratified by CRP. That is, it is possible that there are confounding factors. Therefore, in follow‐up studies, it will be necessary to determine how the *NR1I2* rs3814057, rs7643645, and rs3814055 polymorphisms affect the VRCZ C_0h_ in patients with CRP levels of less than 40 mg/L. Considering the non‐linear pharmacokinetics of VRCZ, we predict that C_0h_ will be found to increase with increasing doses in patients with CRP levels of at least 40 mg/L. Second, because we did not measure the blood concentration of 4‐hydoxyvoriconazole in this study, the effect of the inflammatory response on CYP3A4 activity in the metabolism of VRCZ could not be evaluated. Notably, the inflammatory response has been shown to influence the metabolic activity of both CYP3A4 and CYP2C19^42^, ^44^, ^45^; in fact, Simon et al. used physiologically based pharmacokinetic models to demonstrate that CYP3A4 activity is reduced more than CYP2C19 activity when CRP levels are high.^34^ Therefore, further studies exploring the effects of the inflammatory response on the hydroxylation pathway of VRCZ are needed. Third, the impacts of the *CYP2C19* polymorphism on the side effects and efficacy of VRCZ therapy were not analyzed. The need for clinical trials of VRCZ therapy to determine the usefulness of *CYP2C19* genotyping has long been discussed, but clinical implications of *CYP2C19* polymorphism remain unresolved.^39^ However, our study revealed that the impact of *CYP2C19* polymorphism on VRCZ pharmacokinetic characteristics differed in the presence and absence of an inflammatory response. This result may explain the differences in degrees of the effect of *CYP2C19* polymorphisms on VRCZ C_0h_ noted in previous reports.^46^

## 5 CONCLUSION

Both the *CYP2C19* polymorphism and the status of the inflammatory response affect the *N*‐oxidation pathway, the major metabolic pathway of VRCZ. Therefore, the usefulness of *CYP2C19* genotype analysis in the individualization of VRCZ dosages depends on the state of the patient's inflammatory response. In addition, the *NR1I2* rs3814057, rs7643645, and rs3814055 polymorphisms may also be useful in predicting VRCZ C_0h_.

## DISCLOSURE

Dr. Sakuraba received research funding from Bristol‐Myers Squibb, AbbVie Inc., MSD Inc., DAIICHI SANKYO Co. Ltd., Bayer Schering Pharma, LAVIEPRE Co. Ltd. Dr. Niioka received research funding from Hitachi High‐Tech Co. Ltd., DAIICHI SANKYO Co. Ltd. The remaining authors have nothing to disclose.

## AUTHOR CONTRIBUTIONS

TN conceived the study; NA, HS, TT, KK, NS and MI investigated the study; JN and KU involved in plasma concentration measurement; NA involved in formal analysis; NA wrote the original draft of the manuscript; and KY, HK, and TN wrote, reviewed and edited the manuscript. All authors read and approved the final manuscript.

## ETHICS STATEMENT

The study was conducted in accordance with the Declaration of Helsinki, and the protocol was approved by the Ethics Committee of Hirosaki University Graduate School of Medicine.

## Data Availability

The data that support the findings of this study are available from the corresponding author upon reasonable request.

## References

[1] Scott LJ , Simpson D . Voriconazole: a review of its use in the management of invasive fungal infections. Drugs. 2007;67(2):269‐298. 17284090 10.2165/00003495-200767020-00009

[2] Hicheri Y , Cook G , Cordonnier C . Antifungal prophylaxis in haematology patients: the role of voriconazole. Clin Microbiol Infect. 2012;18(Suppl 2):1‐15. 10.1111/j.1469-0691.2012.03772.x 22409648

[3] Theuretzbacher U , Ihle F , Derendorf H . Pharmacokinetic/pharmacodynamic profile of voriconazole. Clin Pharmacokinet. 2006;45(7):649‐663. 16802848 10.2165/00003088-200645070-00002

[4] Jin H , Wang T , Falcione BA , et al. Trough concentration of voriconazole and its relationship with efficacy and safety: a systematic review and meta‐analysis. J Antimicrob Chemother. 2016;71(7):1772‐1785. 26968880 10.1093/jac/dkw045 PMC4896404

[5] Elewa H , El‐Mekaty E , El‐Bardissy A , et al. Therapeutic drug monitoring of voriconazole in the management of invasive fungal infections: a critical review. Clin Pharmacokinet. 2015;54(12):1223‐1235. 26070947 10.1007/s40262-015-0297-8

[6] Luong M‐L , Al‐Dabbagh M , Groll AH , et al. Utility of voriconazole therapeutic drug monitoring: a meta‐analysis. J Antimicrob Chemother. 2016;71(7):1786‐1799. 27165788 10.1093/jac/dkw099

[7] Park WB , Kim N‐H , Kim K‐H , et al. The effect of therapeutic drug monitoring on safety and efficacy of voriconazole in invasive fungal infections: a randomized controlled trial. Clin Infect Dis. 2012;55(8):1080‐1087. 22761409 10.1093/cid/cis599

[8] Hamada Y , Tokimatsu I , Mikamo H , et al. Practice guidelines for therapeutic drug monitoring of voriconazole: a consensus review of the Japanese Society of Chemotherapy and the Japanese Society of Therapeutic Drug Monitoring. J Infect Chemother. 2013;19(3):381‐392. 23673473 10.1007/s10156-013-0607-8 PMC3682092

[9] Hyland R , Jones BC , Smith DA . Identification of the cytochrome P450 enzymes involved in the N‐oxidation of voriconazole. Drug Metab Dispos. 2003;31(5):540‐547. 12695341 10.1124/dmd.31.5.540

[10] Scholz I , Oberwittler H , Riedel K‐D , et al. Pharmacokinetics, metabolism and bioavailability of the triazole antifungal agent voriconazole in relation to CYP2C19 genotype. Br J Clin Pharmacol. 2009;68(6):906‐915. 20002085 10.1111/j.1365-2125.2009.03534.x PMC2810802

[11] Zhong X , Tong X , Ju Y , et al. Interpersonal factors in the pharmacokinetics and pharmacodynamics of voriconazole: are CYP2C19 genotypes enough for us to make a clinical decision? Curr Drug Metab. 2018;19(14):1152‐1158. 29361899 10.2174/1389200219666171227200547 PMC6635675

[12] Mangal N , Hamadeh IS , Arwood MJ , et al. Optimization of voriconazole therapy for the treatment of invasive fungal infections in adults. Clin Pharmacol Ther. 2018;104(5):957‐965. 29315506 10.1002/cpt.1012 PMC6037619

[13] Desta Z , Zhao X , Shin J‐G , et al. Clinical significance of the cytochrome P450 2C19 genetic polymorphism. Clin Pharmacokinet. 2002;41(12):913‐958. 12222994 10.2165/00003088-200241120-00002

[14] Moriyama B , Obeng AO , Barbarino J , et al. Clinical pharmacogenetics implementation consortium (CPIC) guidelines for CYP2C19 and voriconazole therapy [published correction appears in Clin Pharmacol Ther. 2018 Feb;103(2):349]. Clin Pharmacol Ther. 2017;102(1):45‐51. 27981572 10.1002/cpt.583 PMC5474211

[15] Yanni SB , Annaert PP , Augustijns P , et al. Role of flavin‐containing monooxygenase in oxidative metabolism of voriconazole by human liver microsomes. Drug Metab Dispos. 2008;36(6):1119‐1125. 18362161 10.1124/dmd.107.019646 PMC2737669

[16] Dapía I , García I , Martinez JC , et al. Prediction models for voriconazole pharmacokinetics based on pharmacogenetics: an exploratory study in a Spanish population. Int J Antimicrob Agents. 2019;54(4):463‐470. 31279853 10.1016/j.ijantimicag.2019.06.026

[17] Zeng G , Wang L , Shi L , et al. Variability of voriconazole concentrations in patients with hematopoietic stem cell transplantation and hematological malignancies: influence of loading dose, procalcitonin, and pregnane X receptor polymorphisms. Eur J Clin Pharmacol. 2020;76(4):515‐523. 31932875 10.1007/s00228-020-02831-1

[18] Shirasaka Y , Chaudhry AS , McDonald M , et al. Interindividual variability of CYP2C19‐catalyzed drug metabolism due to differences in gene diplotypes and cytochrome P450 oxidoreductase content. Pharmacogenomics J. 2016;16(4):375‐387. 26323597 10.1038/tpj.2015.58 PMC4775436

[19] Harmsen S , Meijerman I , Beijnen JH , et al. The role of nuclear receptors in pharmacokinetic drug‐drug interactions in oncology. Cancer Treat Rev. 2007;33(4):369‐380. 17451886 10.1016/j.ctrv.2007.02.003

[20] Gautier‐Veyret E , Thiebaut‐Bertrand A , Roustit M , et al. Optimization of voriconazole therapy for treatment of invasive aspergillosis: pharmacogenomics and inflammatory status need to be evaluated. Br J Clin Pharmacol. 2021;87(6):2534‐2541. 33217017 10.1111/bcp.14661

[21] Gautier‐Veyret E , Truffot A , Bailly S , et al. Inflammation is a potential risk factor of voriconazole overdose in hematological patients. Fundam Clin Pharmacol. 2019;33(2):232‐238. 30306637 10.1111/fcp.12422

[22] Vreugdenhil B , van der Velden WJFM , Feuth T , et al. Moderate correlation between systemic IL‐6 responses and CRP with trough concentrations of voriconazole. Br J Clin Pharmacol. 2018;84(9):1980‐1988. 29744898 10.1111/bcp.13627 PMC6089823

[23] Gautier‐Veyret E , Bailly S , Fonrose X , et al. Pharmacogenetics may influence the impact of inflammation on voriconazole trough concentrations. Pharmacogenomics. 2017;18(12):1119‐1123. 28745547 10.2217/pgs-2017-0054

[24] Niioka T , Fujishima N , Abumiya M , et al. Relationship between the CYP2C19 phenotype using the Voriconazole‐to‐Voriconazole N‐oxide plasma concentration ratio and demographic and clinical characteristics of Japanese patients with different CYP2C19 genotypes. Ther Drug Monit. 2017;39(5):514‐521. 28834922 10.1097/FTD.0000000000000441

[25] Veringa A , ter Avest M , Span LFR , et al. Voriconazole metabolism is influenced by severe inflammation: a prospective study. J Antimicrob Chemother. 2017;72(1):261‐267. 27601292 10.1093/jac/dkw349

[26] Encalada Ventura MA , van Wanrooy MJP , Span LFR , et al. Longitudinal analysis of the effect of inflammation on voriconazole trough concentrations. Antimicrob Agents Chemother. 2016;60(5):2727‐2731. 26883707 10.1128/AAC.02830-15 PMC4862487

[27] Naito T , Yamada T , Mino Y , et al. Impact of inflammation and concomitant glucocorticoid administration on plasma concentration of triazole antifungals in immunocompromised patients. Clin Chim Acta. 2015;441:127‐132. 25542532 10.1016/j.cca.2014.12.024

[28] van Wanrooy MJP , Span LFR , Rodgers MGG , et al. Inflammation is associated with voriconazole trough concentrations. Antimicrob Agents Chemother. 2014;58(12):7098‐7101. 25223994 10.1128/AAC.03820-14 PMC4249508

[29] Nivoix Y , Levêque D , Herbrecht R , Koffel JC , Beretz L , Ubeaud‐Sequier G . The enzymatic basis of drug‐drug interactions with systemic triazole antifungals. Clin Pharmacokinetics. 2008;47(12):779‐792. 10.2165/0003088-200847120-00003 19026034

[30] Qi F , Zhu L , Li N , Ge T , Xu G , Liao S . Influence of different proton pump inhibitors on the pharmacokinetics of voriconazole. Int J Antimicrob Agents. 2017;49(4):403‐409. 28159656 10.1016/j.ijantimicag.2016.11.025

[31] Mikus G , Schöwel V , Drzewinska M , et al. Potent cytochrome P450 2C19 genotype‐related interaction between voriconazole and the cytochrome P450 3A4 inhibitor ritonavir. Clin Pharmacol Ther. 2006;80(2):126‐135. 16890574 10.1016/j.clpt.2006.04.004

[32] Klein M , Thomas M , Hofmann U , Seehofer D , Damm G , Zanger UM . A systematic comparison of the impact of inflammatory signaling on absorption, distribution, metabolism, and excretion gene expression and activity in primary human hepatocytes and HepaRG cells. Drug Metab Dispos. 2015;43(2):273‐283. 25480923 10.1124/dmd.114.060962

[33] Li AP , Yang Q , Vermet H , Raoust N , Klieber S , Fabre G . Evaluation of human hepatocytes under prolonged culture in a novel medium for the maintenance of hepatic differentiation: results with the model pro‐inflammatory cytokine interleukin 6. Drug Metab Lett. 2014;8(1):12‐18. 25313020 10.2174/187231280801140929155351

[34] Simon F , Gautier‐Veyret E , Truffot A , et al. Modeling approach to predict the impact of inflammation on the pharmacokinetics of CYP2C19 and CYP3A4 substrates. Pharm Res. 2021;38(3):415‐428. 33686560 10.1007/s11095-021-03019-7

[35] Mbatchi LC , Brouillet JP , Evrard A . Genetic variations of the xenoreceptors NR1I2 and NR1I3 and their effect on drug disposition and response variability. Pharmacogenomics. 2018;19(1):61‐77. 29199543 10.2217/pgs-2017-0121

[36] Prakash C , Zuniga B , Song CS , et al. Nuclear receptors in drug metabolism, drug response and drug interactions. Nucl Receptor Res. 2015;2:101178. 27478824 10.11131/2015/101178 PMC4963026

[37] Murayama N , Imai N , Nakane T , et al. Roles of CYP3A4 and CYP2C19 in methyl hydroxylated and N‐oxidized metabolite formation from voriconazole, a new anti‐fungal agent, in human liver microsomes. Biochem Pharmacol. 2007;73(12):2020‐2026. 17433262 10.1016/j.bcp.2007.03.012

[38] Owusu Obeng A , Egelund EF , Alsultan A , et al. CYP2C19 polymorphisms and therapeutic drug monitoring of voriconazole: are we ready for clinical implementation of pharmacogenomics? Pharmacotherapy. 2014;34(7):703‐718. 24510446 10.1002/phar.1400 PMC4082739

[39] Lee J , Ng P , Hamandi B , Husain S , Lefebvre MJ , Battistella M . Effect of therapeutic drug monitoring and cytochrome P450 2C19 genotyping on clinical outcomes of voriconazole: a systematic review. Ann Pharmacother. 2021;55(4):509‐529. 32772568 10.1177/1060028020948174

[40] Walsh TJ , Moriyama B , Penzak SR , et al. Response to "Pharmacogenetics of Voriconazole: CYP2C19 but Also CYP3A4 Need to Be Genotyped" ‐ The Role of CYP3A4 and CYP3A5 polymorphisms in clinical pharmacokinetics of voriconazole. Clin Pharmacol Ther. 2017;102(2):189 28455946 10.1002/cpt.681

[41] Yamada T , Mino Y , Naito T , et al. Impact of flavin‐containing monooxygenase 3 and CYP2C19 genotypes on plasma disposition and adverse effects of voriconazole administered orally in immunocompromised patients. J Infect Chemother. 2019;25(12):1019‐1025. 31239195 10.1016/j.jiac.2019.05.032

[42] Morgan ET . Impact of infectious and inflammatory disease on cytochrome P450‐mediated drug metabolism and pharmacokinetics. Clin Pharmacol Ther. 2009;85(4):434‐438. 19212314 10.1038/clpt.2008.302 PMC3139248

[43] Gong L , Zhang C‐M , Lv J‐F , et al. Polymorphisms in cytochrome P450 oxidoreductase and its effect on drug metabolism and efficacy. Pharmacogenet Genomics. 2017;27(9):337‐346. 28731962 10.1097/FPC.0000000000000297

[44] Aitken AE , Richardson TA , Morgan ET . Regulation of drug‐metabolizing enzymes and transporters in inflammation. Annu Rev Pharmacol Toxicol. 2006;46:123‐149. 16402901 10.1146/annurev.pharmtox.46.120604.141059

[45] Antachopoulos C , Roilides E . Cytokines and fungal infections. Br J Haematol. 2005;129(5):583‐596. 15916680 10.1111/j.1365-2141.2005.05498.x

[46] Li X , Yu C , Wang T , et al. Effect of cytochrome P450 2C19 polymorphisms on the clinical outcomes of voriconazole: a systematic review and meta‐analysis. Eur J Clin Pharmacol. 2016;72(10):1185‐1193. 27388292 10.1007/s00228-016-2089-y

